# Cytoplasmic and Mitochondrial NADPH-Coupled Redox Systems in the Regulation of Aging

**DOI:** 10.3390/nu11030504

**Published:** 2019-02-27

**Authors:** Patrick C. Bradshaw

**Affiliations:** Department of Biomedical Sciences, James H. Quillen College of Medicine, East Tennessee State University, Johnson City, TN 37614, USA; bradshawp@etsu.edu; Tel.: +1-423-439-4767

**Keywords:** NADPH, aging, redox, lifespan, longevity, mitochondrial, NADP, nicotinamide adenine dinucleotide phosphate, glutathione, thioredoxin, *Drosophila*, *C. elegans*

## Abstract

The reduced form of nicotinamide adenine dinucleotide phosphate (NADPH) protects against redox stress by providing reducing equivalents to antioxidants such as glutathione and thioredoxin. NADPH levels decline with aging in several tissues, but whether this is a major driving force for the aging process has not been well established. Global or neural overexpression of several cytoplasmic enzymes that synthesize NADPH have been shown to extend lifespan in model organisms such as *Drosophila* suggesting a positive relationship between cytoplasmic NADPH levels and longevity. Mitochondrial NADPH plays an important role in the protection against redox stress and cell death and mitochondrial NADPH-utilizing thioredoxin reductase 2 levels correlate with species longevity in cells from rodents and primates. Mitochondrial NADPH shuttles allow for some NADPH flux between the cytoplasm and mitochondria. Since a decline of nicotinamide adenine dinucleotide (NAD^+^) is linked with aging and because NADP^+^ is exclusively synthesized from NAD^+^ by cytoplasmic and mitochondrial NAD^+^ kinases, a decline in the cytoplasmic or mitochondrial NADPH pool may also contribute to the aging process. Therefore pro-longevity therapies should aim to maintain the levels of both NAD^+^ and NADPH in aging tissues.

## 1. NADPH and the Redox Theories of Aging

The reduced form of nicotinamide adenine dinucleotide phosphate, NADPH, protects cells from redox stress and is required for the synthesis of fatty acids, cholesterol, and deoxynucleotides. NADPH is also required for steroid biosynthesis and protects cells from toxic metabolites by providing reducing power to cytochrome P450 enzymes. NADPH levels decrease with age [1,2] due to both aging-related loss of nicotinamide adenine dinucleotide (NAD^+^) [3], a precursor for its synthesis, and to the aging related increase in the [NADP^+^]/[NADPH] ratio that occurs as a result of oxidative stress due to increased mitochondrial electron transport chain dysfunction [4] and inflammation [5]. Therapies that increase NAD^+^ levels combined with therapies that increase NADPH levels could greatly benefit subjects with aging-related disorders. This type of combination therapy has already been shown to be better than either monotherapy alone in an animal model of ischemic stroke [6]. The major objectives of this review are to discuss the mechanisms contributing to the aging-related loss of NADPH, to detail the mechanisms regulating the cytoplasmic and mitochondrial [NADP^+^]/[NADPH], and to give an update on enzymes of the NADPH-powered redox systems that have been identified to modulate lifespan.

The mitochondrial free radical theory of aging (MFRTA) has been a leading theory of aging for nearly 50 years [7] and states that increased levels of mitochondrial reactive oxygen species (ROS) stimulate aging. It therefore suggests that antioxidants and NADPH, which is used for the recycling of many antioxidants, will protect cells from mitochondrial generated ROS to delay tissue and organismal aging. Over the past two decades data has shown that ROS-mediated signaling events in young and healthy organisms can lead to lifespan extension, instead of lifespan decline as was originally proposed by the MFRTA [8]. There are also many experimental results that appear to refute the MFRTA [9]. For example, overexpression of antioxidant enzymes, such as superoxide dismutase (SOD) [10] or catalase [11], or administration of antioxidant supplements, such as vitamins C (ascorbate) [12] or E (tocopherols) [13], do not extend lifespan in several model organisms, although there are exceptions to this statement [14,15,16], the most notable being expression of mitochondrial-targeted catalase extending the lifespan of mice [17].

The fact that many supplemented antioxidants or overexpression of SODs or catalase do not generally extend lifespan could be explained if the activities of the NADPH-fueled redox systems are limiting factors for the protection against lifespan-shortening redox stress and that the activities of SODs and catalase are not limiting. However, it is acknowledged that SOD1 or SOD2 must first detoxify the superoxide produced from the mitochondrial electron transport chain (ETC) to H_2_O_2_ before the NADPH-linked redox systems can detoxify the H_2_O_2_ to water. In addition it is evident that SOD2 plays an essential role in ROS detoxification in newborns due to the death of SOD2 knockout mice shortly after birth [18]. NADPH is required for optimal catalase activity as it binds and stabilizes the catalase tetramer [19]. Antioxidant vitamins such as vitamins C or E that rely upon NADPH to be recycled to their active reduced forms would be expected to provide little longevity benefit in aged organisms where NADPH levels have declined to such an extent to prevent their efficient recycling. The Redox Stress Theory of Aging [20] and the Redox Theory of Aging [21] suggest that lifespan is regulated by redox changes, including alterations in the [NADP^+^]/[NADPH], GSSG/GSH (oxidized glutathione disulfide/reduced glutathione), and oxidized thioredoxin/reduced thioredoxin ratios. These theories also highlight the ability of redox changes to influence aging by not only altering oxidative damage, but by altering cell signaling. Examples of this include the activation of the lifespan-extending transcriptional regulators DAF-16/FOXO and SKN-1/Nrf2 in *C. elegans* by compounds that stimulate ROS production [22,23].

Much data obtained over the past two decades greatly support the MFRTA-derived redox-based theories of aging including the strong negative correlation between the rate of mitochondrial superoxide generation and lifespan in closely related species [24], the strong positive correlation between phospholipid fatty acid saturation levels and lifespan, and the negative correlation between the frequency of cysteine residues in mitochondrial electron transport chain transmembrane spanning regions and lifespan [25]. The higher fatty acid saturation in longer lived species likely evolved to prevent the ROS-mediated oxidation of fatty acid double bonds [26], while the depletion of mitochondrial inner transmembrane cysteine residues likely evolved to prevent thiyl radical formation and potentially lifespan shortening protein crosslinking that can occur when superoxide reacts with protein sulfhydryl groups [27]. The mitochondrial inner membrane is enriched with the phospholipid cardiolipin, which is essential for ETC function and ADP/ATP transport and due to its high degree of fatty acid unsaturation is especially vulnerable to ROS-mediated damage [28]. 

## 2. Loss of NAD^+^ as a Major Cause for Loss of NADPH With Aging

One cause for the aging-related loss of NADPH and increase in oxidative stress with aging is the decrease in the levels of cellular NAD^+^ [3], the immediate precursor for the synthesis of NADP^+^ by NAD^+^ kinases. NAD^+^ levels decline with aging in mammals for several reasons, one of which is the aging-related decrease in the salvage pathway of NAD^+^ synthesis as a result of decreased expression of nicotinamide phosphoribosyl transferase (NAMPT) [29], a committed step in this pathway. There is also an increase in NAD^+^ degradation with aging. The decreased NAD^+^ levels may be a cause of sirtuin protein deacetylase-dependent [30,31] or sirtuin-independent alterations in mitochondrial ETC activity that results in increased ROS production and increased nuclear DNA damage that activates poly-ADP-ribose polymerase (PARP) in several aged tissues including liver, heart, kidney, and lung [32]. This PARP activation together with the aging-related increase in expression and activity of the NAD^+^ and NADP^+^ hydrolyzing enzyme CD38 [33] lead to increased hydrolysis of NAD^+^ and NADP^+^ in aged tissues. CD38 was shown to have greater activity (6-fold lower K_m_ and 2-fold higher V_max_) using NADP^+^ as a substrate than NAD^+^ [34,35]. PARP activation also leads to decreased NADPH levels as PARP inhibits hexokinase, the first enzyme of glycolysis also required for glucose flux into the NADPH-generating pentose phosphate pathway (PPP) [36]. 

In brain, SARM1 is another NADase that contributes to the loss of NAD^+^ under pathological conditions [37]. But whether or not SARM1 is activated in aged brain has yet to be studied in mammals. There are no homologs of CD38 present in the genomes of the aging models *Drosophila* or *C. elegans*, but both genomes encode homologs of PARP and SARM1 [38]. Expression of the *Drosophila* homolog of SARM1 increased during aging or mitochondrial ETC inhibition and was shown to play a role in inducing a pro-inflammatory state [39]. NADP^+^ phosphatase activities, resulting in the degradation of NADP^+^ to NAD^+^, have also been observed in rat liver mitochondrial and Golgi extracts [40,41], but the proteins responsible these activities or any aging-related changes in enzyme activity levels have yet to be identified.

Nematodes and insects, as with other invertebrates, lack NAMPT homologs and the two-step NAD^+^ salvage pathway present in vertebrates, but instead possess a four-step salvage pathway. In this pathway, nicotinamide is first deaminated to nicotinic acid by a nicotinamidase and then the 3-step Preiss–Handler pathway for NAD^+^ salvage synthesis from nicotinic acid is employed [42,43]. In addition, like mammals, *C. elegans* can synthesize NAD^+^ through a de novo pathway from tryptophan [44]. The *Drosophila* genome lacks a 3-hydroxyanthanilic acid dioxygenase gene encoding an enzyme that synthesizes quinolinic acid in the de novo NAD^+^ synthesis pathway [45]. Therefore *Drosophila* appears to strictly rely upon the salvage pathway for NAD^+^ synthesis.

Several lines of evidence suggest that loss of NAD^+^ and NADPH may play a role in the aging of *Drosophila* and *C. elegans*. The NAD^+^/NADH ratio declines with aging in both species [46,47], while in *C. elegans* this loss was driven almost entirely by a decrease in NAD^+^ levels. Since greater than 80% of pyridine nucleotides are bound to proteins and not free in solution [48], the (free) [NAD^+^]/[NADH] as measured by the pyruvate/lactate ratio is a much better functional indicator than measuring total levels. In aging *C. elegans* a loss of (free) [NAD^+^]/[NADH] also paralleled the loss in total NAD^+^/NADH [46]. Addition of NAD^+^ (10 μM–1 mM) [49], nicotinamide (0.2 mM), or nicotinamide riboside (0.5 mM) [50] to the culture medium extended lifespan in *C. elegans*, even though the *C. elegans* genome apparently lacks a nicotinamide riboside kinase gene to convert nicotinamide riboside to nicotinamide mononucleotide. But *C. elegans* possesses homologs to purine nucleoside phosphorylase (PNP) and methylthioadenosine phosphorylase (MTAP) that can likely catabolize nicotinamide riboside to nicotinamide for synthesis of NAD^+^ synthesis through the salvage pathway [51].

The decreased nuclear NAD^+^ levels with aging in mouse muscle lead to stabilization of hypoxia inducible factor-1α (HIF-1α) and decreased c-Myc-induced expression of mitochondrial targeted genes, including mitochondrial transcription factor A (TFAM), required for mitochondrial ETC function [52]. Decreased ETC complex I function with aging increases mitochondrial NADH and decreases mitochondrial NAD^+^ levels, which likely results in decreased phosphorylation of NAD^+^ to NADP^+^ by mitochondrial NAD^+^ kinase 2 (NADK2). Decreased ETC function with aging also reduces the proton-motive force across the mitochondrial inner membrane, which decreases mitochondrial nicotinamide nucleotide transhydrogenase (NNT)-mediated synthesis of NADPH and NAD^+^ from NADP^+^ and NADH in the matrix space.

**Figure 1:**
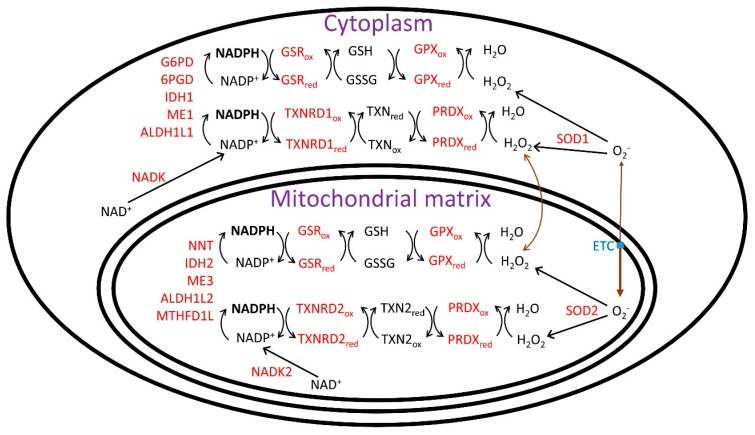
Cytoplasmic and mitochondrial reduced nicotinamide adenine dinucleotide phosphate (NADPH)-linked redox systems. NADK: nicotinamide adenine dinucleotide (NAD^+^) kinase; ETC: electron transport chain; GSR: glutathione reductase; GSH: reduced glutathione; GSSG: oxidized glutathione disulfide; GPX: glutathione peroxidase; TXN: thioredoxin; TXNRD: thioredoxin reductase; PRDX: peroxiredoxin; H_2_O_2_: hydrogen peroxide; O_2_^−^: superoxide; G6PD: glucose-6-phosphate dehydrogenase; 6PGD: 6-phosphogluconate dehydrogenase; IDH: isocitrate dehydrogenase; ME: malic enzyme ALDH: aldehyde dehydrogenase; MTHFD: methylenetetrahydrofolate dehydrogenase; NNT: nicotinamide nucleotide transhydrogenase.

The increase in cytoplasmic and mitochondrial [NADP^+^]/[NADPH] with aging decreases the activities of cytoplasmic and mitochondrial glutathione reductase (GSR) and thioredoxin reductases (cytoplasmic TXNRD1 and mitochondrial TXNRD2), which lead to more oxidized states of the glutathione redox couple (GSSG/GSH) and thioredoxins (cytoplasmic TXN and mitochondrial TXN2), resulting in decreased activities of glutathione peroxidases (GPX) and peroxiredoxins (also called thioredoxin peroxidases). See Figure 1 for an overview of the cytoplasmic and mitochondrial NADPH-linked redox systems. This oxidation of the NADPH-linked redox systems with aging also causes the oxidation of glutaredoxins, ascorbate (vitamin C), and tocopherols (vitamin E) [53] and eventually leads to oxidation of lipids, nucleic acids, and amino acids and the cell and tissue dysfunction that accompanies the aging process. 

## 3. Aging is Associated with Redox Imbalance

All multicellular organisms that have been studied thus far, with the possible exception of extremely long-lived naked mole rats [54], show oxidizing redox changes with aging accompanied by oxidative damage to macromolecules. Redox stress appears to contribute to almost all aging-related disorders including diabetes, heart disease, and neurodegenerative disease. The [NADP^+^]/[NADPH] redox couple is the most important redox couple in the fight against aging-induced cellular oxidation. The redox potentials of the free cytoplasmic and mitochondrial [NADP^+^]/[NADPH] are roughly −400 to −20 mV and are the most negative redox potentials in the cell [55,56]. The cytoplasmic and the mitochondrial [NAD^+^]/[NADH] couples [57], which are roughly 99.9% and 87% oxidized, respectively, in healthy cells [56,58] become more reduced with aging, while the cytoplasmic and most likely also the mitochondrial [NADP^+^]/[NADPH] couple, which are both roughly 99% reduced in healthy cells [56,58] become more oxidized with aging. For example, the energy of the cytoplasmic NADP^+^/NADPH couple became 35 mV less negative with aging corresponding to a 23% loss of brain NADPH levels from 10 to 21 months of age in mice [2]. The initial mechanisms involved in this redox imbalance remain unclear, but at least in the brain the aging-induced redox imbalances may result from the decreased expression of NAMPT and NNT [2].

The GSH/GSSG ratio in cells depends heavily on the [NADP^+^]/[NADPH], but appears not to be in equilibrium due to the moderate expression level of glutathione reductase, the enzyme that links these couples. The GSH/GSSG is approximately 20:1 to 100:1 (95–99% reduced) [59], while during times of oxidative stress this ratio may drop to 5:1 (80% reduced) or even 1:1 (50% reduced) [60]. Cytoplasmic and mitochondrial GSH levels are 1 to 10 mM, two to three orders of magnitude higher than cytoplasmic and mitochondrial [NADPH] [61,62], due in part to the fact that the vast majority of cellular NADP(H) is bound and not free [63]. In addition in rat liver 59% of the total cellular NADP(H) was reported to be present in mitochondria [58], although mitochondria only make up 10–15% of the cell volume. Therefore it may take many hours to days for cells to completely recover from oxidation of GSH if the cells are able to recover at all [64]. 

Opposite to the oxidizing effects of aging on the plasma GSSG/GSH and cystine/cysteine ratios [65], the NADP^+^/NADPH ratio in human plasma has been reported to become more reduced with aging due to increased levels of NADPH, even though NADP^+^ levels declined [66]. Inhibiting cellular export or stimulating cellular uptake of different forms of vitamin B3 (niacin) may be a strategy to maintain cell and tissue NADPH levels with aging. In this regard supplementation with nicotinamide riboside has been shown to be beneficial to increase plasma NAD^+^ levels [67]. No reports of plasma membrane transport of NADP(H) could be found, but intraperitoneal or tail vain injection of NADPH in mice was shown to increase the levels of NADPH in plasma and brain, respectively [68].

In *Drosophila* the NADP^+^/NADPH increased in late adulthood in parallel with the decline in the NAD^+^/NADH [47]. To the best of our knowledge total NADP^+^ and NADPH levels have not been measured in young and aged *C. elegans*. The isocitrate/alpha-ketoglutarate ratio as a measure of the cytoplasmic [NADP^+^]/[NADPH] in young and old worms has also yet to be determined. As only one malic enzyme gene is present in *C. elegans* and it contains a mitochondrial targeting signal [69], measurement of the nematode malate/pyruvate ratio will likely not be indicative of the cytoplasmic [NADP^+^]/[NADPH] as it is in mammals, which possess a cytoplasmic NADP^+^-dependent malic enzyme (ME1) [56]. 

## 4. Reductive Stress Can Influence the Rate of Aging

Although the study of oxidative stress is common, the study of reductive stress is somewhat rare, especially as it pertains to aging. The terms oxidative and reductive stress are somewhat unclear [70] as they do not specifically refer to the [NADP^+^]/[NADPH] or [NAD^+^]/[NADH] couple. Most investigators utilize the term oxidative stress to refer to a state where the [NADP^+^]/[NADPH], GSSG/GSH, and protein cysteine thiols are more oxidized than the normal healthy condition and reductive stress to mean that these redox couples are more reduced than normal.

Quantification of oxidative stress in lysed cell, tissue, or isolated mitochondrial extracts is frequently performed by measurements of GSSG/GSH. The cytoplasmic or mitochondrial [NADP^+^]/[NADPH] is slightly more difficult to measure as one must either use a fluorescent probe in intact cells [71] or measure the ratios of specific metabolites that are in equilibrium with the [NADP^+^]/[NADPH] in lysed cells or tissues [56,58]. Lysed cell measurements of total NADP^+^/NADPH (or NAD^+^/NADH) are not indicative of the free levels as bound nucleotides are released into solution upon lysis [56].

The oxidation state of protein thiols in live cells can be measured using the genetically encoded fluorescent probes roGFP or roGFP2 [72]. High levels of glutaredoxins can equilibrate roGFP or roGFP2 with the redox state of the glutathione pool. Therefore, a roGFP2-glutaredoxin fusion protein was developed and expressed in *Drosophila* to measure the GSSG/GSH with aging [73]. This probe showed a stable, highly reduced cytoplasmic GSSG/GSH with aging, while there was heterogeneous oxidation of the mitochondrial GSSG/GSH in both young and aged fat body, hemocytes, and Malpighian tubules, but a stable reduced GSSG/GSH in young and aged mitochondria from muscles and gut enterocytes [74]. Feeding N-acetylcysteine (NAC) to flies led to reductive stress characterized by slightly decreased cytoplasmic H_2_O_2_ in gut and increased mitochondrial H_2_O_2_ in fat, gut, and Malpighian tubules [74]. 

Further elegant studies using cytoplasmic and mitochondrial targeted roGFP have shown that increasing the reduction of cytoplasmic GSSG/GSH and protein thiols to induce reductive stress as measured by cytoplasmic roGFP leads to production of ROS in mitochondria and oxidative stress as evidenced by oxidation of mitochondrial roGFP [75]. Reducing power in the cytoplasm can be transmitted to the mitochondrial matrix by both the direct transport of GSH into the mitochondrial matrix [76,77] as well as by mitochondrial NADPH shuttles that can transfer NADPH reducing equivalents [78]. A major NADPH shuttle system uses cytoplasmic isocitrate dehydrogenase 1 (IDH1), the mitochondrial tricarboxylate (citrate) carrier, and mitochondrial isocitrate dehydrogenase 2 (IDH2). Once the reducing equivalents are transported into the matrix space, the reduced [NADP^+^]/NADPH] in the matrix space activates glutathione reductase to reduce the GSSG/GSH and activates thioredoxin reductase to reduce thioredoxin. However, once the mitochondrial GSSG/GSH and thioredoxin pools become highly reduced, superoxide and hydrogen peroxide are generated by the mitochondrial glutathione reductase and thioredoxin reductase enzymes as flavins in these two enzymes produce ROS in the absence of their GSSG and oxidized thioredoxin substrates [79].

Adding 2-deoxyglucose to *C. elegans* culture medium or knocking down expression of the glycolytic enzyme glucose-6-phosphate isomerase, *gpi-1*, to inhibit glycolysis led to lifespan extension [23]. The mechanism of lifespan extension has not been fully established, but the authors showed a transient increase in respiration and ROS production following inhibition of glycolysis and showed that lifespan extension could be blocked by supplemented antioxidants [23]. Therefore, they concluded that a ROS signal mediates lifespan extension. However, others have suggested that the addition of antioxidants may have blocked lifespan extension by inducing reductive stress instead of by quenching ROS [80]. It has also been shown that inhibiting glycolysis stimulates metabolic flux through the PPP to increase NADPH production [81], so the lifespan extension by glycolytic inhibition may also have been due in part to a reduced cytoplasmic [NADP^+^]/[NADPH] and increased redox defenses that followed the transient increase in ROS production. Studies using yeast where the glycolytic enzyme triose phosphate isomerase was deleted showed a reduced NADP^+^/NADPH ratio likely due to increased PPP flux, but decreased replicative lifespan [82], perhaps in part due to reductive stress.

In mice reductive stress due to increased NADPH production by the PPP enzyme glucose-6-phosphate dehydrogenase (G6PD) has been shown to be associated with protein aggregation and cardiomyopathy [83]. Reductive stress could also potentially lower the already low levels of cytoplasmic [NADP^+^] (1–100 nM) to decrease the activity of enzymes that rely upon it to drive metabolism. As cytoplasmic oxidative stress appears to be much more prevalent than reductive stress, especially in aged tissues, it does not appear likely that cytoplasmic reductive stress is a cause of mitochondrial oxidative stress in aging tissues or major aging-related diseases.

## 5. Could the Low Levels of Cytoplasmic Glutathione Peroxidase Activity in Long-Lived Species Function in the Preservation of [NADP^+^]/[NADPH] and GSSG/GSH with Aging to Promote Longevity?

Since the redox theories of aging are gaining traction, more detailed studies of the roles of the cytoplasmic and mitochondrial NADPH-glutathione reductase and NADPH-thioredoxin reductase systems in aging are needed. There is also a dearth of knowledge on the mechanisms that cause the mitochondrial ETC of short-lived species to generate more superoxide than that of long-lived species and the mechanisms through which the ETC generates increased levels of superoxide with age [8]. Although compounds that block mitochondrial superoxide production from complex I [84] and complex III [85] without affecting oxidative phosphorylation have recently been identified as potential therapeutics, compounds that stabilize the cytoplasmic and mitochondrial [NADP^+^]/[NADPH] redox couples to maintain redox defense systems with aging are also desperately needed for clinical trials for aging-related diseases.

Naked mole rats have a mean lifespan over 30 years, at least ten times longer than similarly sized mice, and surprisingly have 70-fold less cellular glutathione peroxidase activity than mice [86]. Glutathione peroxidase detoxifies hydrogen peroxide to water through the oxidation of GSH. Therefore, it is possible that increases in specific types of ROS, such as specific types of peroxides in the cytoplasm, are compatible with increased longevity [86]. An alternative to this hypothesis is that the NADPH-thioredoxin reductase-thioredoxin-peroxiredoxin detoxification system for peroxides is upregulated in naked mole rats to compensate for the low activity of the NADPH-glutathione reductase-GSH-glutathione peroxidase system.

The extremely low glutathione peroxidase activity of naked mole rats may even contribute to their extreme longevity through the preservation of reduced cytoplasmic GSSG/GSH and [NADP^+^]/[NADPH]. If this is true, the maintained high energy of the [NADP^+^]/[NADPH] couple can then be used to stimulate longevity through increasing the activities of NADPH-utilizing enzymes such as glutathione reductase and thioredoxin reductase to combat other types of redox stress, such as that induced by peroxynitrite [87], which if left unchecked could decrease longevity. The increased lifespan of mice heterozygous for glutathione peroxidase-4 [88] and the strong inverse relationship between cellular glutathione peroxidase activity and lifespan in seven different rodent species [86] support a role for maintaining reduced cytoplasmic GSSG/GSH and [NADP^+^]/[NADPH] for increased longevity. If only cytoplasmic, but not mitochondrial glutathione peroxidase activity is low in long-lived species, mitochondrial shuttles and glutathione transporters could be used for the increased detoxification of mitochondrial peroxides as a mechanism responsible for the longevity of these species.

Unexpectedly, long lived *Drosophila* strains did not have more GSH than short-lived strains [89]. Likewise, in rodents and in birds there is no correlation between GSSG/GSH in liver and longevity and even a negative relationship was found between liver GSH and longevity [90]. This data suggests that the cytoplasmic NADPH-glutathione reductase-GSH redox system does not limit longevity. As possible compensation for their low GSH levels, liver from naked mole rats contain much higher levels of the cytoplasmically localized thioredoxin reductase (TXNRD1) and major peroxiredoxin (PRDX1) than short-lived guinea pigs [91]. This may be an indication of higher Nrf2 transcriptional activity in naked mole rats [92].

As mentioned above there is also a negative correlation between mitochondrial ETC-produced superoxide production and longevity [8]. Therefore, long-lived species appear to have evolved more efficient ETC machinery that plays an important role in maintaining a reduced intracellular environment. Consistent with this, it was also reported that naked mole rats have greatly decreased levels of proteins for ETC complex I, an important source of ROS, compared to short-lived guinea pigs [91]. However, another report indicated no difference in ETC complex I activity between naked mole rats and mice, but decreased ETC complex II activity in naked mole rats [93]. It was also shown that isolated mitochondria from naked mole rats have a greatly enhanced ability to detoxify hydrogen peroxide compared to those from mice, largely due to higher activity of the mitochondrial NADPH-glutathione reductase-GSH-glutathione peroxidase system [93].

To help resolve some of the current controversies in the field, future studies could focus on measurements of [NADP^+^]/[NADPH] and GSSG/GSH in hypothalamus and white adipose tissue in closely related species with different maximal lifespans. These tissues appear have a greater influence on organismal aging than tissues or cells where past measurements have been made [94,95]. Measurements using mitochondria isolated from these tissues would also be important, as only roughly 10–15% of cellular GSH is present in mitochondria [96]. Importantly NADPH has been shown to bind to the 39 kD subunit of ETC complex I to stabilize the complex [97,98] and ETC complex I activity declines with aging [99,100]. 

## 6. The NADPH-Thioredoxin Reductase Redox System and Aging 

Thioredoxin reductases use NADPH to reduce oxidized thioredoxin. In the cytoplasm TXNRD1 reduces TXN and in mitochondria TXNRD2 reduces TXN2 [101]. Double heterozygous knock out mice (TXN^+/−^/TXN2^+/−^) were surprisingly long-lived [102] suggesting that TXN or TXN2 levels do not limit lifespan, although follow up studies should be performed to determine if compensatory reductions of other redox couples such as the NADP^+^/NADPH or GSSG/GSH are responsible for the increased longevity. Transgenic TXN or TXN2 mice show increased mean, but not maximal lifespan [102,103]. Overexpression of TXN and TXN2 together surprisingly led to decreased lifespan [101] suggesting that redox signaling occurs between the cytoplasmic and mitochondrial compartments. 

Therapies that maintain a reduced [NADP^+^]/[NADPH] in the cytoplasm or mitochondria of aged organisms, but without inducing excessive reductive stress are promising longevity therapies. Longevity studies using mice overexpressing TXNRD1 or TXNRD2 would aid our understanding of the role that these proteins play in mammalian longevity. Mitochondrial TXNRD2 is an especially promising candidate to overexpress or deplete in mice for aging studies because protein levels and activities in fibroblasts positively correlate with longevity across multiple rodent and primate species [104]. Overexpression of the *Drosophila* homolog of TXNRD2, Trxr-2, extended median lifespan [104], while overexpression of the mostly cytoplasmic, but partially mitochondrial *Drosophila* thioredoxin reductase Trxr-1 did not extend lifespan during normoxia [105], but it did during hyperoxia [106]. Further evidence for a role of the mitochondrial TXNRD2-TXN2 system in enhanced longevity is that growth hormone downregulates the expression of these two genes in liver and growth-hormone deficient Ames dwarf mice are long-lived [107].

In rat brain mitochondria that naturally lack catalase, roughly 75% of the hydrogen peroxide generated was shown to be detoxified by the NADPH-TXNRD2-TXN2-peroxiredoxin system, while only 25% was detoxified by the NADPH-glutathione reductase-GSH-glutathione peroxidase system [108]. This result was quite surprising given that GSH is present at roughly 100-1,000 fold higher levels than Txn2 in mitochondria [109]. In addition to reducing oxidized thioredoxin, thioredoxin reductases can reduce dehydroascorbate [110], ubiquinone [111], cytochrome c [112], lipoamide, lipoic acid, [113], and lipid hydroperoxides [114], which also may play a role in the regulation of lifespan. 

Knockdown of *C. elegans* cytoplasmic thioredoxin reductase *trxr-1* [115,116] or mitochondrial thioredoxin reductase *trxr-2* [117] by RNA interference decreased lifespan. However, deletion of *trxr-1* did not affect lifespan [117], while deletion of *trx-2* decreased lifespan at 25 °C, a temperature at the high end of the normal culture range, but not at 20 °C, the most typical culture temperature [117]. TRXR-2 was found to be protective against beta-amyloid toxicity in a *C. elegans* Alzheimer’s disease model [118], while TRXR-1 was found to be protective against 6-hydroxydopamine toxicity in a nematode Parkinson’s disease model [119].

*C. elegans* peroxiredoxin *prdx-2*, homologous to human PRDX1, was required for the lifespan extension that occurs in *daf-2* insulin receptor-deficient worms [120] or when metformin was added to the culture medium [121]. The *prdx-2* gene was also required for the longevity that was induced by exposing *C. elegans* larvae to a 25 °C temperature when the culture temperature was otherwise kept at 15 °C [122]. However PRDX-2 did not become oxidized during the normal aging process in *C. elegans* [123], so its role in lifespan extension could be independent of redox changes.

Perhaps the redox enzyme with the strongest connection with longevity in invertebrates is methionine sulfoxide reductase A (MSRA). During oxidative stress, methionine is oxidized to methionine sulfoxide. MSRA catalyzes the reduction of methionine sulfoxide back to methionine. But MSRA must be reduced by the NADPH-thioredoxin reductase-thioredoxin system to be recycled for use [124]. There are two types of genes that encode isoforms of methione sulfoxide reductase, A and B, that each reduces one of the two isomers of methione sulfoxide. MSRA has been more closely linked with lifespan extension and uses both free methionine sulfoxide and the methionine sulfoxide present in proteins as substrates, while MSRB can only the use methionine-sulfoxide present in proteins [125]. MSRA is present in the cytoplasm, mitochondria and nucleus [126], while there are 3 genes in mammals encoding MSRBs, where the gene products localize to the cytoplasm, mitochondria, nucleus, and ER [126].

Knockdown of the *C. elegans* homolog of MSRA called *msra-1* decreased lifespan [127], likely due to an increase in protein unfolding and proteotoxicity when methionine sulfoxide cannot be repaired. *Msra-1* is induced by the pro-longevity transcription factor DAF-16/FOXO that becomes activated when insulin signaling is blocked. Knockdown of *msra-1* in long-lived *daf-2* insulin receptor mutant worms decreased lifespan. *Drosophila* that simultaneously lack both MSRA and MSRB are short-lived [128], while overexpression of the *Drosophila* homolog of MSRA, mostly in neurons, extended lifespan [129] and stimulated nuclear translocation of dFOXO [130]. However, in mice overexpression of MSRA in either the cytoplasm or mitochondria did not extend lifespan [131]. This may be due to the fact that *Drosophila* MSRA lacks methionine oxidase activity while mammalian MSRA contains methionine oxidase activity [132]. 

## 7. The NADPH-Glutathione Reductase Redox System and Aging 

By analyzing data from multiple rodent and primate species, a positive association between glutathione reductase (GSR) protein levels (and activity) and lifespan in fibroblasts was found [104]. But a negative correlation between brain glutathione reductase activity and lifespan was found in another study [133]. *Drosophila* lacks a glutathione reductase gene and its function is replaced by the two thioredoxin reductase genes [134]. However, *C. elegans* studies of the NADPH-glutathione reductase redox system and aging have been performed. Like mammals *C. elegans* contain only one glutathione reductase gene *gsr-1* that generates both cytoplasmic and mitochondrial protein variants [135]. Lifespan extension of 18% occurred following overexpression [136]. Knockout of *gsr-1* was lethal as it is required for molting [137]. Transgenic overexpression of the cytoplasmic isoform but not the mitochondrial isoform rescued lethality [135]. Knockdown of *gsr-1* by RNAi resulted in decreased lifespan and increased sensitivity to the superoxide generator juglone. Decreased GSR-1 levels may decrease lifespan in part through decreasing autophagy and increasing proteotoxicity [138].

Global low level overexpression of the catalytic subunit of glutamate-cysteine ligase (GCL_c_), the rate-limiting step in glutathione synthesis, or high level overexpression in neurons has also been shown to increase lifespan in *Drosophila* by up to 50%, while overexpression of the regulatory subunit of GCL increased lifespan by up to 24% [139,140]. Administering a recombinant glutaredoxin (small protein reduced by GSH) to *C. elegans* culture medium was shown to extend lifespan dependent upon upregulation of the pro-longevity transcriptional regulator heat shock factor-1 (HSF-1) [141]. Deletion of either of the yeast glutaredoxin genes Grx1 or Grx2 decreased chronological longevity by increasing ROS production that activated Ras-protein kinase A signaling. Therefore, altered redox status and glutaredoxin function modulates lifespan through the activation of longevity regulating signaling pathways. 

Surprisingly, young naked mole rats have been shown to have a more oxidized liver GSSG/GSH than mice [142], even though the naked mole rats live much longer. But the naked mole rats did not show increased levels of oxidative damage with aging as was shown in mice [54]. In mice and rats, important redox couples have been shown to be maintained at distinct, non-equilibrium potentials in different subcellular compartments [143]. The mitochondrial [NADP^+^]/[[NADPH] couple has been estimated to be around −419 mV compared to −400 mV for the cytoplasmic couple [58]. The mitochondrial GSSG/GSH couple was estimated to be between −280 and -330 mV [144,145,146], while the cytoplasmic couple was estimated to be between −200 and −260 mV [145]. So the existing data on GSSG/GSH ratios cannot be used to estimate cytoplasmic or mitochondrial [NADP^+^]/[NADPH]. Therefore, the cytoplasmic and/or mitochondrial [NADP^+^]/[NADPH] may not decline with aging in naked mole rats as they do in mice to drive longevity through maintaining thioredoxin and glutathione reductase activities in one or both of these compartments. Since several markers of oxidative damage, such as urinary isoprostanes (marker of lipid peroxidation) and liver malondialdehyde (marker of lipid damage) are higher in aged naked mole rats than in aged mice [54], naked mole rat redox potential is likely only maintained with aging in one or more specific subcellular compartments and not the entire cell as a whole.

Identifying the redox events that drive the aging process is a high priority. Measurements using isolated mitochondria may be required if the redox events that regulate aging are mostly confined to this organelle [107]. Some data suggest that the mitochondrial [NADP^+^]/[NADPH] redox systems may play a role in longevity regulation, but further studies manipulating the mitochondrial [NADP^+^]/[NADPH], GSSG/GSH, and oxidized thioredoxin/reduced thioredoxin ratios and monitoring lifespan are required to obtain a better understanding of how the activity of the NADPH-powered mitochondrial redox systems influence lifespan.

## 8. Evidence for the Regulation of Longevity by the NADPH-Powered Redox Systems in *Drosophila*

Data supporting the importance of the NADPH-powered redox networks in the regulation of *Drosophila* longevity include overexpression of glucose-6-phosphate dehydrogenase (G6PD), an NADPH-generating enzyme of the oxidative PPP, increasing lifespan by up to 50% [147]. Expression from a neuronal-specific promoter was able to extend lifespan by up to 40%. Longer-lived strains of *Drosophila* were shown to have higher G6PD activity than shorter-lived strains [148]. Knockdown of ribose-5-phosphate isomerase, an enzyme of the non-oxidative portion of the PPP, increased G6PD and NADPH levels and lifespan by over 30% [149]. Overexpression of the NADPH-generating cytoplasmic malic enzyme (Men) [150] extended lifespan in *Drosophila* as well [151,152]. Overexpression of Men, either throughout lifespan or only during larval development, was able to stimulate longevity, while adulthood only expression in the fat body did not extend lifespan. Surprisingly ROS production was increased when overexpression occurred during larval development that increased stress resistance during adulthood. The data suggest that cytoplasmic reductive stress may have been responsible for the increased mitochondrial ROS production during larval development resulting in the lifespan extension. Men, cytoplasmic isocitrate dehydrogenase (Idh), and G6PD have been shown to form a network maintaining cytoplasmic NADPH levels stable with Men having a slightly larger role than either of the other two enzymes [153].

It will be important to determine if overexpression of G6PD only during larval development increases cytoplasmic reductive stress and mitochondrial ROS production during this time period to increase lifespan. Also, it will be important to determine if overexpression of Pug/Pugilist, a cytoplasmic NADPH-generating methylenetetrahydrofolate dehydrogenase enzyme [154,155] extends lifespan. However, these results may be difficult to interpret as targeted overexpression of Nmdmc, a mitochondrial NADH-generating methylenetetrahydrofolate dehydrogenase gene, in the fat body also extended lifespan [156]. Lastly, much information could be gained by determining the effects of overexpression of *Drosophila* mitochondrial NADPH-generating malic enzyme (Men-b) on lifespan. The *Drosophila* genome also contains two other uncharacterized malic enzyme-like genes Menl-1 and Menl-2 whose gene products also likely have a mitochondrial localization [157].

Further data linking NADPH levels with increased lifespan in *Drosophila* include the association between longevity and a specific allele of the cytoplasmic NADPH-generating isocitrate dehydrogenase gene Idh [158] and flies that were selected for a long lifespan showing increased activities of several NADPH-generating enzymes [159]. Interestingly, *Drosophila* lacks homologs to mammalian mitochondrial NADPH-generating isocitrate dehydrogenase (IDH2) and NADPH-generating mitochondrial nicotinamide nucleotide transhydrogenase (NNT), while homologs of both are present in *C. elegans*. However, a proteomics experiment identified the mostly cytoplasmic Idh protein as being present in *Drosophila* mitochondria [160]. Therefore, *Drosophila* likely depends heavily on malic enzyme (Men-b) and the mitochondrial localized fraction of Idh for mitochondrial NADPH production. 

There are seven peroxiredoxin genes in *Drosophila*. Overexpression of three different *Drosophila* peroxiredoxin (thioredoxin peroxidase) genes has been shown to extend lifespan. When expressed in neurons dPrx1/Jafrac1, homologous to mammalian PRDX2, increased lifespan up to 29% [161]. *Drosophila* dPrx5 is endogenously localized to multiple subcellular compartments including the cytoplasm, nucleus, and mitochondria. Global overexpression increased lifespan by more than 30% [162]. Overexpression of dPrx5 specifically in mitochondria also extended lifespan [163]. dPrx3 is exclusively expressed in mitochondria. Although dPrx3 overexpression did not lead to lifespan extension, a dPrx3/dPrx5 double knockout showed premature aging phenotypes [164,165]. dPrx4 is endogenously localized to the ER, and moderate global overexpression or high level overexpression in neurons increased lifespan by greater than 30% [166]. Therefore, the NADPH-thioredoxin reductase-thioredoxin-peroxiredoxin network plays an important role in regulating aging in *Drosophila*. 

## 9. Evidence for the Regulation of Longevity by the NADPH-Powered Redox Systems in Mammals

The study of NADPH synthesis in mammalian cells and tissues is complex as it synthesis is shared among at least 5 cytoplasmic enzymes including G6PD, 6PGD (6-phosphogluconate dehydrogenase), IDH1 (isocitrate dehydrogenase 1), ME1 (malic enzyme 1), and ALDH1L1 (10-formyltetrahydrofolate dehydrogenase) and 5 mitochondrial enzymes NNT, IDH2, ME3 (malic enzyme 3), ALDH1L2 (10-formyltetrahydrofolate dehydrogenase), and MTHFD1L (methylenetetrahydrofolate dehydrogenase 1 like) [167] (Figure 1). ALDH1L1, ALDH1L2, and MTHFD1L play a role in one-carbon and folate metabolism [167], and are an important source of NADPH in proliferating cells [168], where ribonucleotide reductase-mediated deoxynucleotide synthesis utilizes reducing equivalents from the NADPH-thioredoxin reductase-thioredoxin redox system. Overexpression of G6PD increased the mean lifespan of female mice [169].

Levels and activities of NADPH generating enzymes are altered by aging. For example, G6PD levels were shown to decline with aging in mouse brain [170], rat liver [171,172] and human erythrocytes [173], but not lymphocytes [174]. Activity of the PPP enzyme 6PGD was shown to decline by 26% with aging due to lysine oxidation, while malic enzyme activity declined 36% with aging due to histidine oxidation [175]. Activities of IDH1 and IDH2 decreased with aging in rat kidney, but increased with aging in rat testes [176]. Both of these changes were prevented by lifespan-extending calorie restriction (CR). The C57B/6J strain of mice was identified to contain a mutation in the NNT gene, but this strain has a longer lifespan than many other inbred strains. So it appears that the mice are able to compensate for the loss of NNT function and synthesize sufficient NADPH for proper mitochondrial function, at least under normal, non-stressed conditions [177].

Reducing the cytoplasmic [NADP^+^]/[NADPH] may not be beneficial for maintaining a reduced cellular redox environment in all cell types as NADPH oxidase enzymes use NADPH to produce superoxide, and sometimes also hydrogen peroxide [178]. For example, NADPH oxidase Nox2 complexes are present at high levels in macrophages and microglia and function to increase superoxide production. Most NADPH oxidase isoforms are not constitutively active, but require a signal such as increased Ca^2+^ levels for activation. However, the Nox4 isoform is widely expressed, constitutively active, and present in intracellular membranes such as those of mitochondria, ER, and the nucleus. Nox4 produces mostly hydrogen peroxide that is membrane permeable instead of superoxide that is not [179]. Nox4 has a K_m_ for NADPH of 55 μM [180], while human glutathione reductase has a K_m_ for NADPH of ~9 μM [181], and human cytoplasmic thioredoxin reductase (TXNRD1) has a K_m_ for NADPH of 18 μM. Cytoplasmic NADPH has been measured to be at a concentration of 3 μM in transformed cells, while mitochondrial NADPH was measured to be 37 μM in the same cells [61]. Therefore Nox4 activity is likely to be low under normal conditions, but would increase if Nox4 localized to the inner mitochondrial membrane with the active site facing the matrix space where increased levels of NADPH are present. 

Stress increases Nox4 levels and increased Nox4 levels can lead to ETC complex I instability [182]. In addition dominant-negative Nox4 expression caused cytoplasmic reductive stress and increased mitochondrial ROS production, while Nox4 overexpression led to cytoplasmic oxidative stress. Although Nox4 appears to regulate mitochondrial ETC function and ROS production in heart and endothelial cells [183,184], Nox4 knockout mice have a normal lifespan [185]. This is likely due to the low levels of mitochondrial Nox4 in many tissues under normal, healthy conditions [186]. But increased Nox4 expression has been shown to contribute to hydrogen peroxide-induced senescence of endothelial cells [183].

## 10. Evidence for the Regulation of Longevity by the NADPH-Powered Redox Systems in Budding Yeast

When *S. cerevisiae* cells are cultured with glucose as the carbon source the majority of cytoplasmic NADPH was produced by the PPP enzyme Zwf1 (G6PD) and acetaldehyde dehydrogenase Ald6 [187], although the PPP 6PGD enzymes Gnd1 and Gnd2 also contributed to a certain extent [188]. When the yeast cells were cultured using a nonfermentable carbon source instead of glucose, levels of cytoplasmic isocitrate dehydrogenase Idp2 increased and it became an important source of cytoplasmic NADPH [189]. Deletion of Ald6 was shown to increase the chronological longevity of yeast [190].

Most mitochondrial NADPH in yeast is generated by the mitochondrial NADH kinase Pos5. Pos5 also contains an NAD^+^ kinase activity and the Pos5 NAD^+^ kinase activity followed by the activity of the NADPH-generating Ald4 acetaldehyde dehydrogenase, homologous to human ALDH2, was also shown to play a role in mitochondrial NADPH production. Deletion of Ald4 in yeast increased chronological lifespan [191]. Unlike multicellular eukaryotes, yeast mitochondrial malic enzyme Mae1 and mitochondrial NADP^+^-dependent isocitrate dehydrogenase Idp1 only appeared to play minor roles in mitochondrial NADPH generation [187]. Like *Drosophila*, budding yeast lack a homolog to mammalian mitochondrial NNT. In yeast, mitochondrial transporters of NAD^+^ (Ndt1 and Ndt2) provide the vast majority of NAD^+^ for mitochondrial NADP(H) synthesis. Ndt1/Ndt2 double deletion mutants showed decreased mitochondrial and cellular NAD(H) levels and increased chronological lifespan, while Ndt1 overexpression resulted in increased mitochondrial and cellular NAD(H) levels and decreased chronological lifespan [192].

In chronologically aged *S. cerevisiae* cells, NADPH levels and levels of reduced cytoplasmic thioredoxin reductase drop before the majority of cysteine oxidation in proteins and before increased ROS production, while anti-aging dietary restriction (DR) delayed the oxidation of NADPH and thioredoxin reductase and extended lifespan [193]. Therefore, the drop in NADPH levels could be a key driving force in postmitotic cell aging that leads to redox stress. Consistent with this data, deletion of Zwf1 [194] or the cytoplasmic thioredoxin reductase Trr1 decreased chronological longevity when using a strain of the α haploid mating type (MATα), which may be a result of the inverse relationship between thioredoxin reductase activity and the activity of longevity controlling TORC1 kinase [195]. The chronologically short-lived MATα Zwf1 deletion strain had both increased NADP^+^/NADPH and increased NAD^+^/NADH demonstrating that the pro-aging effects of NADPH depletion were dominant over the pro-longevity effects of increased NAD^+^/NADH in this context [194]. However, deletion of the Zwf1 gene increased chronological lifespan when a strain of the a mating type (MATa) was used [196]. Since deletion of NADPH-generating enzymes in yeast generally led to increased longevity, a lifespan-extending signaling pathway appears to be activated by increased NADP^+^/NADPH. Other studies in yeast have shown that the heat shock-induced increase in replicative longevity was due to a transient increase in superoxide levels that signaled for the redirection of metabolic flux from glycolysis to the PPP to increase NADPH synthesis and GSH levels [197].

There are three different NADPH-generating isocitrate dehydrogenase genes in *S. cerevisiae*. Idp1 is mitochondrial, Idp2 is cytoplasmic as mentioned above, and Idp3 is peroxisomal. Deletion of each of these 3 genes increased replicative lifespan in a MATα strain [198,199], while deletion of Idp2 or Idp3 decreased replicative lifespan in a MATa strain. Deletion of Idp1 increased chronological longevity in a MATa strain [196], while deletion of Idp2 decreased chronological longevity in a MATa strain [191]. As a comparison, deletions of Idh1 or Idh2 subunits of the mitochondrial NADH-generating isocitrate dehydrogenase complex led to increased replicative lifespans when using either MATa or MATα strains [198,199]. Since these replicative lifespan analyses were performed on glucose-containing media, where the NADPH-generating isocitrate dehydrogenases do not greatly control NADPH levels, the effects on lifespan are likely due to changes in citric acid cycle flux that may alter the NAD^+^/NADH. Studies like these using a nonfermentable carbon source instead of glucose would be more informative regarding the influence of [NADP^+^]/[NADPH] on yeast replicative lifespan. 

## 11. Evidence for the Regulation of Longevity by NADPH-Powered Redox Systems in *C. Elegans* Nematodes

The most commonly studied model of lifespan extension in *C. elegans* is the *daf-2* insulin receptor-deficient strain [200], which requires the DAF-16/FOXO transcriptional regulator and to a lesser extent the SKN-1/Nrf2 transcriptional regulator for the enhanced longevity [201]. The PPP enzyme GSPD-1/G6PD and the cytoplasmic NADP^+^-dependent isocitrate dehydrogenase IDH-1 likely provide the vast majority of cytoplasmic NADPH in *C. elegans*, while the PPP enzyme T25B9.9/6PGD also likely contributes. Knockdown of *gspd-1* in an *idh-1* mutant strain led to severe growth, locomotion, and molting defects, although individual RNAi-mediated knockdown of *gspd-1* or individual knockout of *idh-1* had no effect on these phenotypes. The growth defect in *gspd-1* and *idh-1* double deficient animals was possibly mediated by alterations in amino acid metabolism as a result of NADPH depletion [202]. Decreased glutamate levels were found in these NADPH-deficient worms that may have been due to decreased glutamate dehydrogenase (GDH-1) activity, which relies upon NAD(P)H as a cofactor. However, GDH-1 is predicted to be localized to mitochondria [69], where GDH-1 would be less affected by GSPD-1 and IDH-1 deficiency than if it were localized to the cytoplasm. Folate metabolism could also contribute to a minor extent to cytoplasmic NADPH levels as *C. elegans* possesses two homologs to human MTHFD1 (NADP^+^-dependent methylenetetrahydrofolate dehydrogenase 1), K07E3.4 and *dao-3*, the latter being transcriptionally regulated by DAF-16/FOXO [203]. 

Long-lived *daf-2* mutants showed increased IDH-1 activity [204], GSPD-1 levels [204,205], and levels of the mitochondrial citrate carrier (K11H3.3), a component of the mitochondrial NADPH shuttle [204]. The increase in IDH-1 activity may be especially important for longevity as we and others have shown that IDH-1 protein levels (as well as cytoplasmic aconitase (ACO-1) levels) decline greatly with aging [46,206]. Therefore, decreased cytoplasmic NADPH levels or decreased mitochondrial NADPH shuttle activity could contribute to aging phenotypes.

There are likely three major enzymes that contribute to mitochondrial NADPH generation in *C. elegans*, nicotinamide nucleotide transhydrogenase-1 (NNT-1), isocitrate dehydrogenase-2 (IDH-2), and malic enzyme (MEN-1) [69,157]. However, the localization of MEN-1 has yet to be confirmed experimentally. *Nnt-1* gene expression is induced mainly in the intestine and neurons by DAF-16 in the long-lived *daf-2* mutant worms [207,208]. Since knockdown of *nnt-1* by RNAi decreased lifespan by 18–20% in both N2 control and *daf-2* mutant worms [207], NNT-1 is likely a major regulator of mitochondrial NADPH levels. NADPH-generating malic enzyme (MEN-1) levels were not altered in long-lived *daf-2* mutants [206].

The increased longevity of *C. elegans daf-2* mutants required increased expression of *icl-1* (*gei-7*) [209], the bifunctional mitochondrial glyoxylate shunt enzyme possessing isocitrate lyase and malate synthase activity [210]. Increased ICL-1 activity decreases flux through mitochondrial IDH-2 and the heterotrimeric (IDHA-1, IDHB-1, and IDHG-1/IDHG-2) mitochondrial NADH-generating isocitrate dehydrogenase. ICL-1 expression levels also correlate with longevity in *eat-2* mutants that undergo dietary restriction and long-lived mitochondrial ETC mutants [211]. However, this correlation could be uncoupled for ETC deficiency, where knockout of the transcriptional regulator *nhr-49* prevented increased *icl-1* expression, but did not prevent increased lifespan [212]. With aging there is a strong decrease in the abundance of the IDHG-2 subunit of the NADH-generating isocitrate dehydrogenase complex [206] and a decrease in the NAD^+^/NADH [46] that would likely increase flux through IDH-2 and counter aging-induced increases in the mitochondrial NADP^+^/NADPH. 

Knockdown of the *C. elegans* mitochondrial citric acid cycle genes aconitase (*aco-2*) [213], isocitrate dehydrogenase subunit *idha-1* [213], or alpha-ketoglutarate dehydrogenase subunits *ogdh-1* [214] or *dld-1* (dihydrolipoamide dehydrogenase) [215] extended lifespan. Knockdown of these enzymes could lead to accumulation of mitochondrial citrate, transport of citrate into the cytoplasm, its conversion into isocitrate (by ACO-1) and then into α-ketoglutarate by IDH-1 to reduce cytoplasmic [NADP^+^]/[NADPH] and increase lifespan.

*C. elegans* GSPD-1/G6PD may be a very important source of NADPH for removing old cuticle for molting during larval development [137]. Consistent with its important role in NADPH synthesis a *gspd-1* knockout is inviable [216]. Unexpectedly, knocking down each of four cytoplasmic PPP enzymes 6PGD (T25B9.9), transaldolase (*tald-1*), transketolase (*tkt-1*) [217], or *gspd-1* (G6PD) [205] or knocking down *idh-1* [213] extended lifespan. The lifespan extension may have been a result of upregulated expression of compensatory NADPH generating enzymes, through ROS-mediated activation of SKN-1/Nrf2 and/or through activation of the mitochondrial unfolded protein response (UPR^mt^) [217,218]. UPR^mt^ is a stress response pathway that frequently, but not always, associates with lifespan extension [219] and is associated with activation of the transcriptional regulators ATFS-1 (ATF4, ATF5, and CHOP in mammals), DVE-1, and SKN-1/Nrf2 [220]. During larval development ATFS-1 was activated in ETC complex I *nuo-6* mutants that resulted in DAF-16/FOXO and HIF-1 activation and increased longevity [221].

In *Drosophila,* mutations that decreased Idh and malic enzyme (Men) activities were accompanied by a compensatory upregulation of G6PD activity [153,222], while in mice decreased G6PD activity leads to a compensatory increase in IDH1 levels [223]. G6PD is also allosterically activated by NADP^+^ [224,225]. Perhaps due to these responses, knockdown of *C. elegans idh-1* [213], *gspd-1* (G6PD) [205] or T25B9.9 (6PGD) [217] led to lifespan extension. Knockdown of the PPP gene *tald-1*, beginning from the L1 larval stage extended lifespan, but not when knockdown was initiated during adulthood [217]. In contrast, knockdown of *gcs-1*, the rate limiting step of glutathione synthesis, had no effect on lifespan when initiated at the L1 larval stage, but extended lifespan when initiated at the L4 larval stage [226]. Low levels of the glutathione depleting compound diethyl maleate increased lifespan through a DAF-16 and SKN-1 dependent mechanism, but high diethyl maleate levels decreased lifespan [226]. These compensatory effects that modulate longevity partly driven by DAF-16 and SKN-1 make aging studies where the cytoplasmic redox state is oxidized in young individuals difficult to interpret unless detailed redox measurements are made in parallel.

A *C. elegans* RNAi screen identified 41 genes including *gspd-1*, other PPP genes, and glutathione reductase (*gsr-1*) as the strongest inducers of the SKN-1/Nrf2 target gene *gcs-1* [218]. SKN-1 is activated by oxidative stress and regulates expression of hundreds of genes. It decreases expression of insulin-like peptides to activate DAF-16/FOXO [227]. Together DAF-16 and SKN-1 induce several NADPH-generating enzymes and antioxidant enzymes. However, DAF-16 and SKN-1 activity greatly decline with aging [228], which may contribute to the increased NADP^+^/NADPH with aging. Juglone, an oxidant that induces robust activation of the SKN-1 reporter *Pgst-4::gfp* in young worms was completely ineffective in activating the reporter by day 10 of the lifespan [228], while *daf-2* knockdown failed to extend lifespan when initiated at day 8 of adulthood or later [229]. Consistent with their important roles in longevity, *skn-1* or *daf-16* knockdown can slightly decrease normal lifespan [230].

Compensatory responses to NADPH depletion also likely occur in humans as G6PD deficiency is associated with long life [231]. As NADPH is a known activator of the class I histone deacetylases (HDACs) HDAC1 and HDAC2 [232], part of this compensatory response may involve decreased activity of class I HDACs, which extends lifespan in model organisms [233]. 

Some data do not support a role for NADPH levels to be limiting for lifespan. For example, deletion of the mitochondrial *nnt-1* in *C. elegans* increased the GSSG/GSH ratio, but did not decrease lifespan [234]. However, unexpectedly as referred to above, RNAi knockdown of *nnt-1* in *C. elegans* decreased lifespan by 18% [207]. The reasons for the different effects between knockout and knockdown of *nnt-1* on lifespan are not known. Completely knocking out *nnt-1* may lead to greater ROS production and activation of SKN-1 and DAF-16-mediated protective pathways than incompletely knocking it down. Not only does ROS-mediated SKN-1/Nrf2 activation induce expression of NADPH-generating enzymes to partially compensate for decreased NADPH levels [235], it also increases expression of other antioxidants including superoxide dismutase [236], catalase [237], and enzymes of glutathione metabolism [238]. Activation of SKN-1 by knocking out its endogenous inhibitor WDR-23 extended lifespan, but unexpectedly increased the GSSG/GSH ratio [239], yet another example of how the cellular GSSG/GSH ratio is not always predictive of lifespan. Determining the effects of SKN-1 activation or DAF-16 activation on cytoplasmic and mitochondrial [NADP^+^]/[NADPH] may help to decipher the mechanisms through which each extends lifespan.

## 12. Calorie Restriction May Extend Lifespan In Part through the NADPH-Driven Redox Systems

Calorie restriction (CR) extends lifespan in the vast majority of eukaryotic organisms from yeasts to humans [240] and studies using mice have shown that the methionine restriction (MR) induced by the CR diet provides much of this longevity effect through decreasing mitochondrial ROS production [241] and increasing hydrogen sulfide production through the transulfuration pathway [242]. Flux from methionine degradation through the transulfuration pathway results in cysteine production. The majority of cysteine from the diet is oxidized to cystine and requires NADPH oxidation to reduce it to cysteine once it is taken up by cells [243]. Reduction of the extracellular cystine/cysteine has been shown to increase the intracellular levels of NADPH and GSH [244]. Therefore, use of cystine for cysteine synthesis decreases cellular NADPH levels and use of the transulfuration pathway for cysteine production conserves NADPH. Somewhat paradoxically, CR or MR increases expression of transulfuration pathway enzymes [245]. Long-lived Ames dwarf mice also show increased transulfuration pathway activity [246]. The increased cysteine production through this pathway likely leads to decreased cellular cystine uptake, decreased oxidation of NADPH to reduce cystine, and stabilization of the [NADP^+^]/[NADPH] with aging leading to lifespan extension. 

Methionine synthesis in yeast requires 3 NADPH molecules. Studies have shown that a Zwf1 (G6PD) mutant yeast strain becomes a methionine auxotroph as it is not able to produce the NADPH needed for methionine synthesis [247]. Genes of methionine metabolism appear to be co-regulated with PPP enzymes [248]. Methionine auxotrophs show extended replicative [249] and chronological longevity, while methionine supplementation decreases chronological longevity [250,251]. MR may provide an optimal level of cellular NADPH for longevity by minimizing methionine oxidation to methionine sulfoxide, which requires NADPH oxidation to repair, while maintaining high enough levels of methionine where methionine synthesis does not deplete NADPH levels. Arginine and lysine synthesis in yeast also require NADPH and synthesis becomes impaired when NADPH levels are low [252,253]. But, lysine auxotrophy does not increase chronological longevity [250].

Some of the largest consumers of NADPH include the enzymes for the biosynthesis of fatty acids, cholesterol, steroids, and deoxyribonucleotides [48]. During CR, AMP kinase (AMPK) becomes activated [254], prominently in the hypothalamus [255]. AMPK phosphorylates acetyl-CoA carboxylase to inhibit fatty acid synthesis [255]. This inhibition of fatty acid synthesis leads to the activation of fatty acid beta-oxidation (except in neurons that lack these enzymes) to provide cellular energy and prevent a futile cycle of fatty acid synthesis and breakdown. The inhibition of fatty acid synthesis preserves NADPH levels and contributes to the increased levels of redox defenses associated with the longevity benefits of CR. Stimulating AMPK using the AMP analog AICAR (5-Aminoimidazole-4-carboxamide ribonucleotide) increased the cellular NAD^+^/NADH to activate the NAD^+^-dependent sirtuin deacetylase SIRT1 [256], which deacetylates and activates the master regulator of mitochondrial gene expression PGC-1α to stimulate mitochondrial biogenesis and increase expression of SOD2 and catalase. Blocking mitochondrial fatty acid uptake with etomoxovir blocked the change in NAD^+^/NADH [256]. The reduced [NADP+]/[NADPH] induced by AMPK activation functions together with the increased expression of SOD2 and catalase to maintain redox status in the presence of increased mitochondrial ETC function.

The effect of the anti-aging CR diet in rodents on antioxidant systems has been reviewed [257]. Overall, the majority of studies showed no overall change in mitochondrial ROS production or antioxidant enzyme levels by CR. However, GSH was increased, and the GSSG/GSH and oxidative damage were decreased in the vast majority of studies supporting the redox stress theory of aging. The Nrf2 transcriptional regulator was not required for lifespan extension by CR [258]. Two major mechanisms likely drive the reduced GSSG/GSH and decreased oxidative damage in CR mice. These include the CR-mediated increase in mitochondrial SIRT3 levels and the increased activity of FOXO transcriptional regulators due to decreased insulin signaling during CR. SIRT3 has been shown to deacetylate and activate mitochondrial IDH2 to increase mitochondrial NADPH production [259], while the FOXO3 transcriptional regulator increases PPP flux to increase cytoplasmic NADPH [260]. Neural progenitor cells from FOXO3 knockout mice showed increased NADP^+^/NADPH and GSSG/GSH ratios due to decreased glucose uptake and PPP pathway activity. Furthermore SIRT1 overexpression, which mimics the increased expression of SIRT1 during CR in muscle and white adipose tissue [261], stimulated the formation of a FOXO3/PGC-1α complex that was shown to induce expression of the mitochondrial redox factors TXNRD2, TXN2, peroxiredoxin 3 (PRDX3), and peroxiredoxin 5 (PRDX5) [262].

CR was also shown to blunt the aging-related loss of cytoplasmic TXNRD1 and TXN protein levels in rat kidney [263]. In contrast, in rat cardiac and skeletal muscle aging and CR had no effect on TXNRD1 levels, while CR decreased the aging-related loss of TXN [264]. In muscle, mitochondria-localized TXNRD2 decreased with aging, while CR blunted this effect. Muscle mitochondrial TXN2 levels increased with aging with no effect of CR [264]. CR was also shown to decrease the skeletal muscle levels of TXNIP, an inhibitor of TXN, to enhance TXN function [265]. Extension of lifespan by DR in yeast also required the peroxiredoxin Tsa1 and increased expression of a sulfiredoxin gene to decrease the oxidation of Tsa1 with aging [266]. In *C. elegans*, lifespan extension by DR required the thioredoxin *trx-1* [267]. Global protein cysteine levels were shown not to be oxidized during aging in *Drosophila*, although 24 hours of fasting caused a major oxidation of protein cysteines [268], most likely by decreasing PPP flux and NADPH generation. However, the use of tissue-specific redox probes for H_2_O_2_ and GSSG/GSH were able to measure an aging-induced oxidation of the cytoplasm of midgut enterocytes [74]. Surprisingly long-lived *chico* (insulin receptor substrate) heterozygotes showed increased, not decreased midgut oxidation.

Some portion of the disease delaying effects of CR may be mediated by the increased levels of ketone bodies that occur as a result of the CR diet [55], as a ketogenic diet increased the mean lifespan of mice [269,270]. Excitingly, exogenous ketone body supplementation partially mimicked the protective redox changes induced by CR in the brain (i.e. reduced GSSG/GSH [257]) as exogenous ketone bodies were able to reduce the cytoplasmic [NADP^+^]/[NADPH] in the cerebral cortex of 3XTgAD Alzheimer’s mice, but not in their hippocampus [271]. However, oxidative damage as measured by protein carbonyl levels (2,4-dintrophenylhydrazine reactivity) and lipid peroxidation (4-hydroxynonenal levels) was decreased by ketone body treatment in the hippocampus, but not the cortex of these animals. The data suggest that hippocampal NADPH was oxidized to prevent oxidative damage in this region of the brain and this oxidation of NADPH was the likely mechanism through which no ketone body-induced change in the [NADP^+^]/[NADPH] was found. However, the cerebral cortex of these mice may have lacked high enough NADPH-coupled redox enzyme levels to utilize the reduced [NADP^+^]/[NADPH] for the prevention of oxidative damage. The exogenous ketone treatment also resulted in increased cytoplasmic [NAD^+^]/[NADH] in both brain regions similar to CR and increased mitochondrial [NAD^+^]/[NADH] only in the hippocampus [271]. 

## 13. Regulation of Aging by NADPH and the Circadian Clock

It has been clearly demonstrated in several model organisms that robust circadian rhythms are associated with longevity and that disrupting circadian oscillations in humans and other models is associated with aging and aging-related disorders [272,273]. It was found that 43% of mouse protein coding genes oscillated in expression in a circadian manner in at least one of twelve tissues studied [274], while the levels of roughly half of the mRNAs for these proteins oscillated [275] suggesting post-transcriptional events that regulate the circadian changes in the other half of these proteins. The NAD^+^/NADH and the NADP^+^/NADPH ratios oscillate over a 24 hour cycle [276,277] due in part to circadian regulation of NAMPT [278,279], NADK [280], and the PPP [281,282]. Therefore, loss of circadian rhythms could play an important role in the decreased NAD^+^ [3] and NADPH [1,2] levels observed in aged tissues. Disruption of the PPP through the administration of 6-aminonicotinic acid was described to lengthen the circadian period in one study [282], while disruption of the PPP with the inhibitors diphenyleneiodonium (DPI) or dehydroepiandrosterone (DHEA) was described to alter the phase and amplitude of the circadian changes without affecting the period in another [281]. To limit the potential side effects of small molecule inhibitors, future studies could express the highly specific NADPH-oxidizing enzyme triphosphopyridine nucleotide oxidase (TPNOX) [283], an engineered mutant of *Lactobacillus brevis* NADH oxidase, in cells and target it to the mitochondria or to the cytoplasm to determine the compartment-specific role of changes in the [NADP^+^]/[NADPH] on the circadian clock. 

CR may extend longevity in part by restoring the loss of circadian rhythms. This may occur through the increased NAD^+^/NADH and reduced NADP^+^/NADPH conferred by CR, as dimerization of the circadian clock proteins CLOCK and BMAL1 is regulated by redox state [284]. Also, NADPH binds the circadian transcriptional regulator NPAS2 and modulates its dimerization with BMAL1 and DNA binding of the heterodimer [285,286]. In addition, circadian oscillations, especially in genes involved with metabolism, occur in *C. elegans* [287], *Drosophila* S2 cells [288], and budding yeast [289], organisms that lack essential components of the well-characterized circadian clock, suggesting an evolutionarily ancient uncharacterized circadian system regulating metabolism. It will be important to determine the influence of changes in the [NAD^+^]/[NADH] and [NADP^+^]/[NADPH] on this ancient circadian system.

## 14. NAD^+^ Kinases as Possible Regulators of NADPH Levels and Longevity

The human cytoplasmic NAD^+^ kinase (NADK) was identified in 2001 [290] and has been fairly well-characterized since then, including its regulation by Ca2+/calmodulin-dependent protein kinases [291] and the control of expression by the circadian clock [280]. Mice lacking NADK are not viable [292]. The *Drosophila melanogaster* genome contains two homologous genes, while the *C. elegans* genome contains no homologs of human NADK. So up until the year 2012, it was unknown how *C. elegans* obtained NADP(H). In 2012 a pivotal discovery of the human mitochondrial NAD^+^ kinase (NADK2) was made [293,294]. The *C. elegans* genome contains two homologous uncharacterized genes, while the fruit fly genome contains one. Recently, studies using genetically encoded iNap fluorescent NADPH sensors have verified the exchange of NADPH equivalents between the cytoplasmic and mitochondrial compartments in mammalian cells [61]. Overexpression of cytoplasmic NADK resulted in a 4-fold increase in cytoplasmic [NADPH] and a 57% increase in mitochondrial [NADPH].

Like other eukaryotes, yeast rely on cytoplasmic and mitochondrial NAD(H) kinases to provide NADP(H). The two yeast cytoplasmic NAD(H) kinases, Yef1 and Utr1 appear to prefer NAD^+^ as a substrate unlike mitochondrial Pos5, which prefers NADH [187]. In the filamentous fungus *Podospora anserine,* loss of a mitochondrial NADH kinase gene extended lifespan by apparently inducing expression of an ETC alternative oxidase that decreased ROS production [295].

## 15. Pharmacological Modulation of Cellular Redox Status to Prevent Cellular Senescence and Delay Aging

Methylene blue is a redox active compound that has been demonstrated to delay cellular senescence [296] and showed promise in a phase II clinical trial for Alzheimer’s disease [297]. It has been shown to accept electrons from NADH or reduced flavins and pass them to ETC complex III [298] bypassing superoxide generation from ETC complexes I or II [299]. Presumably these redox properties are responsible at least in part for the many other protective cellular responses including inhibition of the NLRP3 inflammasome [300], activation of the proteasome [301], and stabilization of HSF1 [302]. Methylene blue treatment was shown to extend the lifespan of *C. elegans* [303] and female mice [304], but not *Drosophila* [305].

A screen of 2684 compounds identified the redox-active compounds violuric acid (VA) and 1-naphthoquinone-2-monoxime (N2N1) as the top hits that delayed cellular senescence [306]. These compounds were then shown to increase the lifespan of *C. elegans* and mice. VA greatly reduced the level of cellular protein disulfides by facilitating electron transfer from NADH (and NADPH to a lesser extent) to GSSG to increase GSH and the cellular NAD^+^/NADH. VA also facilitated the transfer of electrons from NADH to H_2_O_2_ to form water, but did not transfer electrons from NADH to cytochrome c. N2N1 was shown to pass electrons from NADH (and NADPH to a lesser extent) to CoQ_10_ and to cytochrome c with the NQO1 (NAD(P)H: quinone oxidoreductase 1) redox system as a major target. Consistent with these results, overexpression of NQO1 together with another NADH-oxidizing redox protein, cytochrome b_5_ reductase 3 (CYB5R3), extended lifespan in mice and mimicked some phenotypes of CR [307]. However, invertebrates such as *C. elegans* lack the NQO1 system, so the longevity effects of N2N1 and methylene blue in nematodes may be due to stimulation of electron transfer from NADH to ETC complex III or to the *C. elegans* CYB5R3 homologs HPO-19 or T05H4.4. Increasing the NAD^+^/NADH ratio is likely key to the longevity effects of these compounds, but VA may also stabilize the NADP^+^/NADPH to provide longevity benefits. Compounds that facilitate the selective transfer of electrons from NADH to GSSG, H_2_O_2_, oxidized thioredoxin, and other electron acceptors could increase the NAD^+^/NADH with aging and would therefore be compounds for the potential treatment of aging-related disorders. Compounds that facilitate the selective transfer of electrons from NADPH to these electron acceptors may also be beneficial at low levels, but at high levels they also have the potential to increase the [NADP^+^]/[NADPH] leading to oxidative stress.

## 16. Summary and Future Perspectives

Evidence from model organisms strongly suggests that redox changes influence the rate of aging. However, both oxidizing and reducing changes can lead to increased longevity making redox measurements in both cytoplasmic and mitochondrial compartments especially important in when determining the mechanism for lifespan extension. In *S. cerevisiae* and *C. elegans*, deleting or knocking down NADPH-generating enzymes increased longevity more frequently than decreasing it, likely due to the active stress response pathways that have evolved in young cells to compensate for the loss of NADPH. Manipulations that lead to a reduction of the [NADP^+^]/[NADPH] in the cytoplasm are especially linked with increased longevity. But reducing the redox potential of the cytoplasm frequently leads to reductive stress characterized by mitochondrial oxidation and ROS production. Mitochondrial ROS production and oxidation is linked with increased longevity when it occurs in young organisms capable of mounting robust longevity enhancing stress responses. The theory that mild reductive stress in the cytoplasm during development leads to increased mitochondrial superoxide production and lifespan extension should be tested in model organisms.

Another clear trend in the literature is the protective effects of the mitochondrial NADPH-linked redox systems. It would be helpful to explore potential molecular mechanisms behind the positive association between mitochondrial thioredoxin reductase activity and increased longevity. This could be accomplished in part through overexpression studies and measurements of mitochondrial superoxide or hydrogen peroxide production, GSSG/GSH, and [NADP^+^]/[NADPH] measurements. However, overexpression of mitochondrial thioredoxin reductase on its own would be expected to have little effect on longevity, as was verified in studies using *Drosophila* [104], without a corresponding reduction of the mitochondrial [NADP^+^]/[NADPH] that drives its enzymatic activity. Since little is known regarding the effects of a more reduced mitochondrial redox environment on longevity, future important experiments will be to overexpress a mitochondrial NADPH-generating enzyme, such as Men-b or mitochondrial targeted Idh in *Drosophila*, to determine the effects of reduced mitochondrial [NADP^+^]/[NADPH] on lifespan.

## Abbreviations

ADP: adenosine diphosphate; AICAR, 5-aminoimidazole-4-carboxamide ribonucleotide; ALDH1L1, cytoplasmic 10-formyltetrahydrofolate dehydrogenase; ALDH1L2, mitochondrial 10-formyltetrahydrofolate dehydrogenase; AMP, adenosine monophosphate; AMPK, AMP kinase; ATP, adenosine triphosphate; CR, calorie restriction; CYB5R3, cytochrome b5 reductase 3; DAF-16, dauer formation-16; DAF-2, dauer formation-2; DHEA, dehydroepiandrosterone; DPI, diphenyleneiodonium; DR, dietary restriction; ER, endoplasmic reticulum; ETC, Electron transport chain; FOXO, forkhead box O; G6PD, glucose-6-phosphate dehydrogenase; GCL, glutamate-cysteine ligase; GDH-1, glutamate dehydrogenase-1; GPI-1, glucose-6-phosphate isomerase-1; GPX, glutathione peroxidase; GSH, glutathione; GSSG, glutathione disulfide; GSR, glutathione reductase; HDAC, histone deacetylase; HSF-1, heat shock factor-1; IDH1, isocitrate dehydrogenase 1 (cytoplasmic); IDH-1, *C. elegans* isocitrate dehydrogenase-1; IDH2, isocitrate dehydrogenase 2 (mitochondrial); IDH-2, *C. elegans* isocitrate dehydrogenase-2; Idh, *Drosophila* isocitrate dehydrogenase; ICL-1, isocitrate lyase-1; K_m_, Michaelis constant; MATa, haploid mating type a; MATα, haploid mating type α; Men, *Drosophila* cytoplasmic malic enzyme; Men-b, *Drosophila* mitochondrial malic enzyme; MEN-1, *C. elegans* malic enzyme; ME1, malic enzyme 1; ME3, malic enzyme 3; MFRTA, Mitochondrial free radical theory of aging; MSRA, methionine sulfoxide reductase A; MSRB, methionine sulfoxide reductase B; MTAP, methylthioadenosine phosphorylase; MTHFD1L, methylenetetrahydrofolate dehydrogenase 1 like; mV, milliVolt; mM, milliMolar; N2N1, 1-naphthoquinone-2-monoxime; NAC, N-acetylcysteine; NAD^+^, nicotinamide adenine dinucleotide (oxidized form); NADK, NAD^+^ kinase; NADK2, mitochondrial NAD^+^ kinase; NADH, nicotinamide adenine dinucleotide (reduced form); NADP^+^, nicotinamide adenine dinucleotide phosphate (oxidized form); NADPH, nicotinamide adenine dinucleotide phosphate (reduced form); NAMPT, nicotinamide phosphoribosyl transferase; NQO1, NAD(P)H: quinone oxidoreductase 1; NNT, nicotinamide nucleotide transhydrogenase; Nox4, NADPH oxidase 4; Nrf2, Nuclear factor (erythroid-derived 2)-like 2; PARP, poly-ADP-ribose polymerase; 6PGD, 6-phosphogluconate dehydrogenase; PNP, purine nucleoside phosphorylase; PPP, pentose phosphate pathway; PRDX, peroxiredoxin; ROS, reactive oxygen species; SARM1, Sterile alpha and TIR motif containing 1; SKN-1, skinhead-1; SOD, superoxide dismutase; TFAM, mitochondrial transcription factor A; TPNOX, triphosphopyridine nucleotide oxidase; TRXR-1, *C. elegans* cytoplasmic thioredoxin reductase; TRXR-2, *C. elegans* mitochondrial thioredoxin reductase; TXN, cytoplasmic thioredoxin; TXN2, mitochondrial thioredoxin, TXNRD1, cytoplasmic thioredoxin reductase; TXNRD2, mitochondrial thioredoxin reductase; UPR^mt^, mitochondrial unfolded protein response; VA, violuric acid; V_max_, maximal velocity.
